# The early diagnostic value of serum neopterin and cartilage oligomeric matrix protein for osteoarticular changes among brucellosis patients at an early period

**DOI:** 10.1186/s13018-018-0932-9

**Published:** 2018-09-04

**Authors:** Zhi-jun Zhao, Qiang Li, Li Ma, Ji-quan Li, Li-qing Xu

**Affiliations:** Qinghai Institute for Endemic Disease Prevention and Control, Xining, 811602 Qinghai China

**Keywords:** Brucellosis, Cartilage oligomeric matrix protein, Neopterin, Biomarker

## Abstract

**Background:**

Brucellosis is an important public health problem in China. Brucellosis can cause many osteoarticular changes, especially chronic brucellosis. Brucellosis presents various diagnostic difficulties because it mimics many other diseases. Because of the poor sensitivity and ability of X-ray image, it is necessary to find a good method of early diagnosis for osteoarticular changes among brucellosis patients at an early period.

The aim of this study was to find early changes biomarkers for osteoarticular changes among brucellosis patients at an early period and provide a better understanding of the osteoarticular changes in this disease at an early stage.

**Methods:**

Sixty-one cases of brucellosis patients at an early period (within 6 months) and 67 cases of volunteers were selected and divided into brucellosis vs. control groups. Serum samples were detected with serological tests for brucellosis, including rose bengal plate test (RBPT), agglutination test (SAT), and IgG and IgM with ELISA. Meanwhile, cartilage oligomeric matrix protein (COMP) and neopterin (NPT) levels in all samples were measured simultaneously with ELISA. The data were analyzed using SPSS 17.0 software.

**Results:**

Together with the clinical examination, epidemiological investigation, and serological tests of RBPT, SAT, and IgG and IgM with ELISA, the patients from brucellosis group all were diagnosed as brucellosis cases at an early period, while the results of RBPT, SAT, and IgG and IgM with ELISA from the healthy control group were negative. Comparing with the healthy control, the medians of serum NPT and COMP in the patients group were 9.26 ng/l, 1.70 ng, respectively, which were higher than that of the healthy control group with significant differences (*Z* = 5.512, 3.614, all *p* = 0.000).

**Conclusion:**

The serum NPT and COMP levels of brucellosis patients at an early period were increased. The serum NPT and COMP levels might be the indicator biomarker for osteoarticular changes of human brucellosis at an early stage.

## Background

Brucellosis is a zoonotic disease with worldwide distribution. In China, brucellosis cases have been reported in all 32 provinces [1], and human brucellosis was endemic in 25 of 32 provinces (or autonomous regions) of mainland China [2]. The incidence rates of human brucellosis were positively associated with the density of sheep and goats [3]. Although the mortality of brucellosis is low, morbidity rates are much higher. At present, brucellosis can be regarded as an important public health problem in China.

With a broad spectrum of clinical manifestations, brucellosis presents various diagnostic difficulties because it mimics many other diseases [4]. Brucellosis can result in many osteoarticular changes, such as sacroiliitis, peripheral arthritis, spondylitis, and bursitis [5]. According to the diagnostic criteria of human brucellosis in China (WS269-2007), most of the chronic brucellosis patients present the osteoarticular changes. Because of the poor sensitivity and early diagnostic ability of X-ray image, thus, osteoarticular changes became the main clinical manifestations among chronic brucellosis. So, it is necessary to find early osteoarticular changes among human brucellosis at an early period for early prevention and control.

Cartilage oligomeric matrix protein (COMP), a non-collagen biomarker for cartilage degradation, is a 524 kD glycoprotein found in articular cartilage, ligament, meniscus, synovial membrane, and tendon [6, 7]. Serum levels of COMP in patients with osteoarthritis (OA) and rheumatoid arthritis (RA) were elevated [8]. COMP could predict cartilage loss [9]. COMP could be used as a prognostic and diagnostic indicator and as a marker of the disease severity and the effect of treatment [10].

Neopterin (NPT) is released from activated T lymphocytes [11]. NPT is used to elevate the extent of cellular immune activation of cellular immune activation [12]. NPT is a useful marker in the follow-up of brucellosis patients and for evaluating the efficacy of therapy [13].

Human brucellosis could cause musculoskeletal changes with high prevalence. Musculoskeletal changes of most of the chronic brucellosis patients are expressed irreversible, and the effective methods of delaying musculoskeletal changes among these chronic brucellosis patients are drug therapy and other therapy methods, so it is very important for preventing musculoskeletal changes at the early stage of human brucellosis. We hypothesized that there were cartilage and immune changes at the early stage of human brucellosis before osteoarticular changes occur among these brucellosis patients, which were expressed by X-ray image or clinical manifestations. The body fluid changes could screen the osteoarticular changes among these patients; thus, we can prevent musculoskeletal changes of human brucellosis by serum biomarkers detection at an early stage in this disease. The aim of this study was to find biomarkers for the osteoarticular changes of human brucellosis and provide a better understanding of this disease.

## Methods

This study was in compliance with the ethical principles in the Declaration of Helsinki of the World Medical Association and was approved by the Ethics Committee of the Qinghai Institute for Endemic Disease Prevention and Control. All subjects signed an informed consent form.

### The diagnostic criteria of human brucellosis

According to the diagnostic criteria of human brucellosis in China (WS269-2007), the patients were diagnosed as brucellosis based on epidemiological, clinical, serological or bacteriological data. The main clinical manifestations are fever, hyperhidrosis, joint and muscle ache, weakness, and headache. The clinical types are acute period (within 3 months after infection, high fever, other symptoms, and high serum titer), sub-acute period (among 3–6 months, low fever, and positive serum titer), and chronic period (above 6 months after infection, normal body temperature and positive serum titer).

The serological tests included rose bengal plate test (RBPT), serum agglutination test (SAT), and detection of IgG and IgM with enzyme-linked immunosorbent assay (ELISA). PBRT is a screen test, while SAT is to further clarify the diagnosis for human brucellosis. IgG and IgM are useful for identifying infectious time.

### Study subjects

This case-control study was carried out in the Qinghai Institute for Endemic Disease Prevention and Control. All the study population were not diagnosed with rheumatic fever, rheumatic arthritis, typhoid fever, osteoarthritis, paratyphoid fever, and tuberculosis after clinical, serum, and X-ray image examination at the hospital, which these diseases were similar to human brucellosis at the clinical manifestation. The main clinical manifestations of some of these persons were fever, weakness, and headache, and some of them have no clinical changes; however, due to special occupation and living environment, they were again checked in the Qinghai Institute for Endemic Disease Prevention and Control from 2013 to 2016. A total of 128 persons were included in this study. Sixty-one patients, who were diagnosed with early brucellosis and without drug therapy (within 6 months), were divided into a patients group. Through matching, another 67 healthy persons were divided into a healthy control group with random sampling, whose serum RBPT and SAT were negative. The patients group included 41 males and 20 females, which the age ranged from 21 to 82 years, while the healthy control group included 44 males and 23 females, which the age ranged from 23 to 77 years. The patients group and the healthy control group had the same production or living environment.

Venous blood samples were collected from all study subjects and were centrifuged at 3000 rpm for 15 min. The serum samples were separated and stored at − 80 °C until assayed. NPT and COMP levels in all the serum samples were quantitatively measured using a commercial ELISA kit, according to the kit procedure (Shifeng Co. Ltd., Shanghai). The results were expressed as ug/l.

### Statistical analysis

The means and standard deviations were calculated for age. The data of serum tests of brucellosis were expressed with frequency, and data of IgG and IgM were analyzed with the chi-square test. The data of NPT and COMP were analyzed for skewed distribution. The median of variables showing skewed distribution was compared by the nonparametric test. A *p* value < 0.05 was considered to be statistically significant. Data were analyzed using SPSS 17.0 software (SPSS, Chicago, IL, USA).

## Results

Based on epidemiological, clinical, serological, or bacteriological data, 61 brucellosis patients were classified as early patients. The average age of brucellosis patients was 39.41 ± 10.51 years; meanwhile that of the healthy controls was (40.31 ± 12.52 years. There was not a significant difference in gender comparison among the two groups (Table 1).

**Table 1:** The age condition of objective person (unit: year)

Gender	Patients	Healthy control	*t*	*p*
Male	39.69 ± 9.98 (41)	42.07 ± 13.70(44)	0.912	0.364
Female	39.00 ± 12.09(20)	36.97 ± 9.27(23)	0.620	0.538
Total	39.41 ± 10.51(61)	40.31 ± 12.52(67)	0.444	0.651

Sixty-one brucellosis patients were RBPT positive, and then these patients were confirmed by SAT (the titer ≥ 1:100). All the brucellosis patients were positive for RBPT and SAT (Table 2). Among them, 98.36% (60/61) and 37.70% (23/61) of brucellosis patients were positive for IgG and IgM, respectively, with a significant difference between the two antibodies (χ^2^ = 51.174, *p* = 0.000), meaning that most of the patients have been infected with *Brucella melitensis* for at least 3 months. The healthy control groups were also detected with serum tests of RBPT, SAT, and IgG and IgM, and all the results of the serum tests were negative.

**Table 2:** Results of traditional serological diagnostic methods of human brucellosis

Groups	RBPT(+)	SAT(+)	IgG	IgM
Patients	61	61	60	23
Healthy control	0	0	0	0

All the 128 persons were tested the serum levels of NPT and COMP (Table 3). The median levels of serum NPT and COMP in the patients group were 9.26 μg/l and 1.70 μg/l, respectively, while the median levels of serum NPT and COMP in the healthy control group were 0 and 0.79 μg/l, respectively. Comparing the results of serum NPT and COMP with rank sum test of the nonparametric test, there were significant differences among the two groups (*Z* = 5.512, 3.614, all *p* = 0.000).

**Table 3:** Results of serum NPT and COMP of objective person (unit: ug/l)

Biomarkers	Gender	Patients				Healthy control				*p*
		Median	Range	25%Q	75%Q	Median	Range	25%Q	75%Q	
NPT	Female	8.53	6.64–37.42	7.29	12.43	0	0–26.72	0	2.54	0.006
	Male	9.30	5.90–63.49	7.34	12.82	0	0–64.30	0	13.07	0.000
	Total	9.26	5.90–63.49	7.44	13.37	0	0–64.30	0	6.51	0.000
COMP	Female	2.31	0.87–17.45	1.21	4.04	0.67	0–6.56	0.44	3.33	0.006
	Male	1.62	0.64–22.25	1.22	2.62	0.83	0–12.76	0.53	4.67	0.012
	Total	1.70	0.64–22.25	1.22	2.76	0.79	0–12.76	0.49	3.97	0.000

*Q* interquartile range,

## Discussion

Generally, the clinical presentation and laboratory detection of human brucellosis-associated epidemic findings are typical, and the diagnosis is not difficult [14]. Osteoarticular change is the most common complication of brucellosis [15], and this variation may be related to the differences in pathogenicity of the species [16], so the diagnosis of osteoarticular changes in this disease is often delayed. Currently, there is no biochemical test for the detection of early diagnosis in osteoarticular changes of human brucellosis. We detected the serum COMP and NPT of patients infected with brucellosis to find the biomarkers for osteoarticular changes in this disease.

The serum COMP levels were stable during the daytime in patients with osteoarthritis and in those with rheumatoid arthritis [17]. Among different types of early arthritis (including self-limiting disease), raised serum COMP levels could be used as an indicator of ongoing cartilage involvement [18]; meanwhile, serum COMP levels were also sensitive to OA disease progression, so serum COMP levels could be used as a prognostic marker of OA [19].

NPT could be used as a nonspecific biomarker in clinical practice in many diseases, such as infectious diseases and osteoarthritis. NPT is associated with oxidative stress and indicative of a pro-inflammatory immune status [20]. NPT is a sensitive marker for the activity of inflammatory RA or OA [21]. In healthy adults, the average concentration of serum NPT was less than 2.23–2.46 μg/l, and its cutoff value was 3.04 μg/l [22]. Elevated NPT concentrations were useful for signalizing the activation of the human immune system.

There were many research on the serum changes of NPT and COMP levels in musculoskeletal diseases [17–22]; however, these research were rarely found in human brucellosis, which also resulted in osteoarticular changes. In this study, the median levels of serum NPT and COMP in brucellosis patients at an early period were elevated, which indicated the changes of the immune system and cartilage among the people diagnosed as brucellosis. There were changes of serum NPT and COMP levels among human brucellosis with/without different osteoarticular changes at an early period. Especially, we first found an early change of cartilage by determining the serum COMP level, which provides a new method for early diagnosis of osteoarticular changes in this disease, indicating that there were cartilage lesions at the early stage in this disease. COMP could be used as a biomarker of osteoarticular changes in human brucellosis. The higher levels of serum NPT might be an inflammatory reaction caused by brucellosis, in accordance with the changes of cell immunity in this disease. Although serum NPT was researched on human brucellosis once by reference index [13], the serum NPT on the mechanism of human brucellosis was rare. At the same time, OA and RA patients could happened the same change of serum NPT, thus serum NPT also might be an inflammatory biomarker of indicating the inflammatory changes and osteoarticular changes in human brucellosis. More studies are needed to find the effect of NPT and COMP on these diseases.

Meanwhile, there were a few limitations in the study. First, there is a lack of osteoarticular changes data of X-ray images among these patients at an early period. Second, more biomarkers for reflecting osteoarticular changes were not included in this study, and different types of biomarkers could explain the osteoarticular changes more reasonably. Third, brucellosis patients could happened the changes in these biomarkers at an early period, but we also should know the changes in these biomarkers at a different stage. Meanwhile, some in vitro studies with chondrocytes were needed to explain its mechanism deeply, and we should constitute a future endeavor in this field.

## Conclusion

Through this study, we found that brucellosis patients can happened the changes of serum NPT and COMP levels, which might be related with the immune system and cartilage lesions. The serum NPT and COMP levels of brucellosis patients at an early period were increased. The serum NPT and COMP levels might be the indicator biomarker for osteoarticular changes of human brucellosis at an early stage. Further research was needed, such as the relationship between the serum biomarkers and the human brucellosis with/without the osteoarticular changes and the association the early patients with/without the osteoarticular clinical symptoms with different types of biomarkers. Meanwhile, some in vitro studies with chondrocytes were needed to explain its mechanism deeply, and we should constitute a future endeavor in this field.

## Abbreviations

COMP: Cartilage oligomeric matrix protein
NPT: Neopterin
RBPT: Rose bengal plate test
SAT: Agglutination test
